# Validity, reliability and minimal detectable change in the sit-to-stand test for synchronous and asynchronous tele-assessment in post-COVID-19 condition

**DOI:** 10.7717/peerj.20211

**Published:** 2025-11-27

**Authors:** Juliane Machado Marques, Matheus Brasiliano da Paz, Rodrigo Rodrigues Gomes Costa, Frederico Ribeiro Neto

**Affiliations:** 1 Neurological Rehabilitation, SARAH Network of Rehabilitation Hospitals, Brasília, DF, Brazil; 2 Paralympic Sports Department, SARAH Network of Rehabilitation Hospitals, Brasília, DF, Brazil

**Keywords:** COVID-19, Muscle strength, Pandemics, Physical fitness, Rehabilitation, Telerehabilitation

## Abstract

**Background:**

Individuals with symptoms of long-term COVID-19 (coronavirus disease 2019) who presented mild infection without needing invasive ventilatory support require rehabilitation and performance and monitoring evaluations. The 1-minute sit-to-stand test (STS) is used to assess individuals with COVID-19 and might be an alternative for remote use in this population.

**Objective:**

The current study aimed to validate the synchronous and asynchronous STS tele-assessment in individuals with post-COVID-19 condition to analyze the inter-evaluator reliability of the asynchronous STS, and identify the relationship between the participants’ self-reported asynchronous STS results and those of the evaluator.

**Methods:**

Thirty-eight men and women with post-COVID syndrome who did not require invasive ventilator support were included in this study. The participants were assessed using STS in-person (STS-IP), synchronous (STS-S), and asynchronous (STS-A) tele-assessments. The participants also self-reported the total executed repetitions (STS-SR). The main outcomes were the number of repetitions performed in the STS-IP, STS-S, STS-A, and STS-SR. To verify STS-A reliability, the total repetitions registered between evaluators were compared.

**Results:**

STS-IP showed significant correlations and lower total repetitions compared to STS-S and STS-A. STS-A repetitions were significantly lower compared to STS-SR; however, a significant correlation was observed. The STS-S and STS-A showed minimal detectable change values of 6.6 and 10.5, respectively. In terms of reliability, there were no significant differences in total repetitions or errors found between evaluators’ assessments of STS-A.

**Conclusion:**

The study demonstrated good and moderate evidence of validity for synchronous and asynchronous remote STS assessments, respectively, highlighting the impact of the assessment protocol on STS performance interpretation. Asynchronous STS presented high reliability.

## Introduction

In late 2019, an infectious disease caused by SARS-CoV-2 was diagnosed, the coronavirus disease 2019 (COVID-19) (Núñez-Cortés et al., 2021; Van Kessel et al., 2022). COVID-19 is a viral and systemic infection that causes severe acute respiratory syndrome and might be aggravated by several risk factors, such as advanced age, sex (male), and the presence of comorbidities (*e.g.*, diabetes, obesity, and cardiovascular diseases). Individuals diagnosed with COVID-19 also presented long-term symptoms (fatigue, joint or muscle pain, general weakness, difficulty in breathing during exertion), defined by the World Health Organization (WHO), as a post-COVID-19 condition or long-term COVID-19 condition (Ali & Kunugi, 2021; Gobbi et al., 2021; Sallis et al., 2021; Soriano et al., 2021; Van Kessel et al., 2022). Until July 2023, there were more than 765 million confirmed cases of COVID-19, with mortality in more than 6.9 million infected people (World Health Organization, 2025).

In the face of a global framework, with great social and health impacts in the medium and long term, there is a need for health monitoring during and after the pandemic period (Crispo et al., 2021). Functional and physical performance assessments become important at this time, as alternatives to measure cardiorespiratory outcomes for individuals with post-COVID-19 condition, such as oxygen desaturation on exertion (Belli et al., 2020; Simonelli et al., 2020). Thus, the 1-minute sit-to-stand test (STS) has been used in health services due to its simplicity, easy applicability, and feasibility (Baricich et al., 2021; Belli et al., 2020; Gephine et al., 2020; Johnsen et al., 2021; Martin et al., 2021; Núñez-Cortés et al., 2021; Paneroni et al., 2021; Zampogna et al., 2021). Several studies have studied rehabilitation outcomes in COVID-19 patients with severe infections (during the hospital stay) (Cheval et al., 2021; Hoyois et al., 2021; Martin et al., 2021; Núñez-Cortés et al., 2021; Tuzun et al., 2021). However, individuals with symptoms of long-term COVID-19, who presented mild infection without ventilatory support, the most prevalent COVID-19 population, require rehabilitation and, consequently, performance and monitoring evaluations (Van Kessel et al., 2022).

Given this scenery of social isolation, many individuals worldwide have been deprived of access to health-related interventions, monitoring, or assessment. Thus, alternative strength and functional tele-assessments are needed for individuals with post-COVID-19 condition that overcome distance limitations. Studies have shown that exercise programs performed remotely are viable and effective in different populations, especially in individuals who need to maintain physical performance due to functional losses (Bryant, Fedson & Sharafkhaneh, 2020; Gomes Costa et al., 2021; Gonzalez-Gerez et al., 2021; Martin et al., 2021; Middleton et al., 2020; Núñez-Cortés et al., 2021; Pinto et al., 2020). Likewise, tests and evaluations are increasingly being performed remotely (Gushikem et al., 2022; Winters-Stone et al., 2020), considering the importance of establishing objective measures for the development of an adequate training program, patient follow-up, and management definitions. In addition, adapted assessments performed remotely, synchronously, and asynchronously, have shown good correlation with in-person tests (Bryant, Fedson & Sharafkhaneh, 2020; Gushikem et al., 2022; Winters-Stone et al., 2020).

Recently, Rees-Punia, Rittase & Patel (2021) validated the asynchronous self-reported STS in 30 s in individuals diagnosed with cancer. In individuals with COVID-19 or post-COVID-19 condition, the 1-minute STS is commonly used (Baricich et al., 2021; Belli et al., 2020; Johnsen et al., 2021; Núñez-Cortés et al., 2021; Paneroni et al., 2021; Zampogna et al., 2021) due to its simplicity and the possibility of verifying oxygen desaturation after physical exertion (Núñez-Cortés et al., 2021). Evidence supports the validity of the test, with significant correlations reported with lower limb strength (Ritchie et al., 2005; Zanini et al., 2015), distance in the six-minute walk test (Ozalevli et al., 2007; Vaidya et al., 2016; Zanini et al., 2015), and physiological responses such as dyspnea (Ozalevli et al., 2007; Vaidya et al., 2016), blood lactate (Canuto et al., 2010), and heart rate (Segura-Ortí & Martínez-Olmos, 2011). It also demonstrates good test-retest reliability (intraclass correlation coefficients—ICC—ranging from 0.80 to 0.98) (Radtke et al., 2016; Ritchie et al., 2005; Segura-Ortí & Martínez-Olmos, 2011) and responsiveness, with a minimal detectable change between 1.9 and 5.4 repetitions (Radtke et al., 2016; Segura-Ortí & Martínez-Olmos, 2011; Vaidya et al., 2016). However, the test duration and the population evaluated may influence the validity of the results. Additionally, not all methods of tele-assessment of the STS (synchronous, asynchronous, and self-reported asynchronous) have been thoroughly studied, and the validity of different tele-assessments has not been compared to determine which is the most appropriate for the professional clinical reality.

Thus, the present study aimed to (1) validate the synchronous and asynchronous STS tele-assessment in individuals with post-COVID-19 condition who did not require ventilator support; (2) analyze the inter-evaluator reliability of the asynchronous STS; and (3) identify the relationship between the participants’ self-reported asynchronous STS repetitions and the results obtained by evaluators. The initial hypotheses were: (1) the synchronous and asynchronous STS are valid tele-assessments; (2) the inter-evaluator reliability will present a high and significant correlation; and (3) the participants’ self-reported asynchronous STS will present a significant and positive correlation with the results of the asynchronous STS.

## Materials and Methods

### Study design

This cross-sectional study adhered to the STROBE checklist guidelines and involved adult men and women with post-COVID-19 condition, recruited from a specialized hospital’s post-COVID-19 rehabilitation program. Participants initially completed an in-person 1-minute sit-to-stand test. Within 48 to 72 h, they repeated the test remotely in either a synchronous or asynchronous format, with the order randomized. Participants self-reported their repetition count through a messaging app in the asynchronous modality. After another 48 to 72 h, they completed the remaining test format. The primary outcome was the number of repetitions performed in each modality, and inter-evaluator reliability was assessed in the asynchronous recordings based on five predefined video criteria.

### Subjects

Thirty-eight men and women with post-COVID-19 condition who did not require ventilator support during treatment for COVID-19 and who participated in the rehabilitation process of a Network Centre of Rehabilitation Hospitals were recruited (Table 1). Data were collected from March 2022 to May 2023. The SARAH Rehabilitation Hospital Network Ethics Committee approved the study (protocol n. 5.448.177), and all participants provided written informed consent.

**Table 1:** Participant demographic data. Age, body mass, height, and physical activity are presented as mean (standard deviation), and the time since COVID-19 diagnosis and fatigue severity scale as median (25th and 75th percentiles).

** **	**Total**	**Female**	**Male**
**n**	38	31	7
**Age (years)**	47.8 (±12.2)	46.9 (±11.3)	51.7 (±16.1)
**Body mass (kg)**	79.1 (±18.1)	75.8^*^ (±16.9)	93.7 (±17.5)
**Height (cm)**	161.8 (±8.6)	159.2^*^ (±7.2)	173.0 (±3.5)
**Physical activity (h/week)**	2.3 (±2.1)	2.2 (±2.1)	2.6 (±2.2)
**Time since COVID-19 diagnosis (months)**	11.8 (5.4–19.2)	11.7 (5.3–19.1)	14.3 (11.8–19.5)
**Fatigue Severity Scale**	50.5 (40.0–57.8)	53.0^*^ (43.0–58.0)	21.0 (19.0–43.5)

**Notes.**

* Significant difference from the male group (*p* ≤ 0.05).

Inclusion criteria were: (1) previously confirmed SARS-CoV-2 PCR with symptoms of the post-COVID-19 condition, such as general fatigue, respiratory fatigue, and muscle or joint pain that are not explained by alternative diagnoses (The Lancet, 2020; World Health Organization, 2025); (2) adult individuals (from 18 years old). Participants were excluded if they: (1) had motor injuries in the upper or lower limbs or disabling pain that could hamper test performance; (2) used orthosis or prosthesis; (3) had oxygen saturation below 90% or a reduction of 4% or more after the in-person test (Briand et al., 2018; Núñez-Cortés et al., 2021); (4) had systolic blood pressure values greater than 180 mmHg or diastolic blood pressure values greater than 100 mmHg (Núñez-Cortés et al., 2021) or (5) required invasive ventilatory support during treatment for COVID-19.

### Procedures

**Figure 1:**
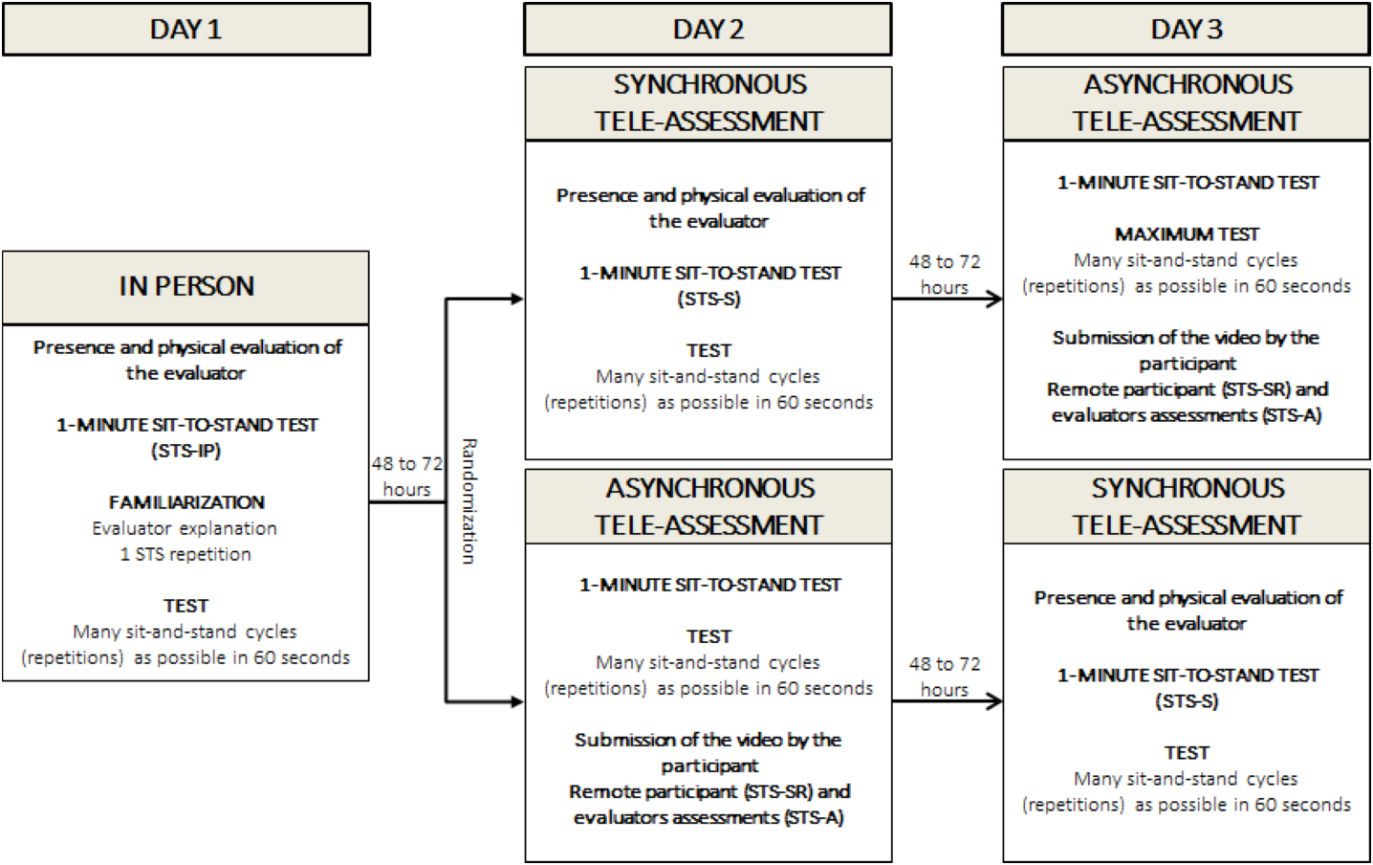
Protocol for STS (synchronous and asynchronous strength tele-assessments).

On day one, the participants were advised of the procedures, received standardized instructions about the STS execution technique, and underwent a clinical assessment. The in-person STS (STS-IP) was conducted on the same day by an evaluator experienced in performing the test. After a 48- to 72-hour interval the STS was performed: (1) synchronously: using video calling guided by the evaluators (STS-S) or; (2) asynchronously: the participants performed the STS at home and sent the test video to the evaluators (STS-A), as well as the self-reported total of executed repetitions (STS-SR). After another 48- to 72-hour interval, participants repeated the STS test in the format (synchronous or asynchronous) that had not yet been performed. The order of the synchronous and asynchronous tests was randomized (https://www.randomizer.org/) (Fig. 1). After each test, the participants’ rating of perceived exertion (RPE) was measured using the Borg scale (Borg & Kaijser, 2006). Additionally, data regarding the participants’ weekly hours of physical activity and their scores on the Fatigue Severity Scale were collected for sample description purposes (Mograbi et al., 2018).

#### 1-minute sit-to-stand test

The test was performed using a chair of standard height (46 cm) without armrests, positioned against a wall. The participants were not allowed to use their hands/arms to push the seat of the chair or their body (arms crossed over the chest during the movement execution) (Bohannon & Crouch, 2019; Ribeiro Neto et al., 2023). The evaluator explained the test execution technique and instructed the participants to complete as many sit-to-stand cycles (repetitions, reps) as possible in 60 s at a self-paced speed. Prior to the test, one repetition was performed for familiarization and possible corrections. The participants started the test in a sitting position, with their back to the chair, and were required to stand up until full extension of the hips and knees, and then sit down until touching their hip on the chair. The evaluator counted each complete movement cycle (sitting, standing, and returning to the sitting position) aloud and informed the participant when 10 s remained. No verbal stimulus or encouragement was given during the test, but reminders were provided for the participant to stand up fully (Bohannon & Crouch, 2019; Ribeiro Neto et al., 2023).

The STS-S and STS-A order was random and occurred at 48–72 h intervals. The STS-S was performed by video call using WhatsApp (WhatsApp software, version 2.21.14.24, 2021, Android, WhatsApp Inc., Menlo Park, CA, USA) and was conducted by a different evaluator from the STS-IP so that the initial result was blinded. During the STS-A, the participant filmed the test and self-reported the number of repetitions in the test (STS-SR). The video was sent by WhatsApp to two independent evaluators for assessment. The total number of repetitions reported by the participants was compared to the correct repetition number registered by the evaluators. The video was analyzed by two independent evaluators (FRN and MB) who did not participate in the STS-IP, based on five predefined criteria: (1) chair size (approximately between 44 and 48 cm, or at the knee line); (2) arms crossed at the sternum without using them to assist in the test; (3) hip touching the seat of the chair; (4) full hip and knee extension; and (5) total test time. The mean of the analysis was considered, and when the difference was greater than 5%, a third evaluator evaluated the result. On the day of the STS-A, participants received a notification to remind them of the data collection procedures.

### Main outcomes

The main outcomes were the number of repetitions performed in the STS-IP, STS-S, STS-A, and STS-SR. To verify reliability in the STS-A, the total repetitions registered between evaluators were compared considering five video criteria: (1) chair size; (2) arms crossed at the sternum without using them to assist in the test; (3) hip touching the seat of the chair; (4) full hip and knee extension; and (5) total test time.

### Statistical analysis

A sample size of 38 individuals was calculated considering an *a priori* correlation with a two-tailed distribution, a moderate effect size (*f* = 0.45), *α* = 5%, and a power of 85% (1 − *β*) (Faul et al., 2007; Faul et al., 2009). The Shapiro–Wilk test was used to assess the data normality assumptions. Descriptive data are presented as mean and standard deviation for parametric data and as median and interquartile range (25th and 75th percentiles) for nonparametric and ordinal variables.

The ICC, Bland Altman plot with linear regression to assess the relationship between bias and the magnitude of measurements, and paired samples *T*-test were used to correlate and compare: (1) STS-IP with STS-S and STS-A repetitions and; (2) STS-A with STS-SR repetitions. The ICC was calculated with a Two-Way Model, Type: Agreement, and Unit: Single Measure, following the recommendations by Koo & Li (2016) and Koo & Li (2016). The ICC was classified based on Cicchetti’s standards: below 0.40—the level of clinical significance is poor; 0.40 to 0.59—fair; 0.60 to 0.74—good; 0.75 to 1.00—excellent (Cicchetti, 1994). Confidence intervals of 95% (95% CI) were used between comparisons. Cohen’s *d* effect size (ES) was calculated and classified in the following manner (Cohen, 1988): trivial (*d* lower than 0.10); small (*d* between 0.10−0.29); moderate (*d* between 0.30−0.49); large (*d* between 0.50−0.69); very large (*d* between 0.70−0.89); and perfect (*d* of 0.90 or greater). The standard error of measurement (SEM) (SEM = SD ×$\sqrt{1}$-ICC) and the minimal detectable change (MDC) (MDC = SEM ×1.96 ×$\sqrt{2}$) for the STS-S and STS-A were also calculated (Weir, 2005). Briefly, the SEM reflects absolute measurement error (response stability) (Weir, 2005), and the MDC provides an objective threshold that can be used to determine whether values obtained are beyond measurement variability (*i.e.,* the smallest difference that can be accurately measured). According to COSMIN guidelines (Mokkink et al., 2012), SEM and MDC are typically estimated from test–retest data collected using the same method under stable conditions. In the present study, however, these indices are applied to quantify agreement between STS-S and STS-A. Additionally, *a posteriori* analysis using a linear mixed-effects model was performed with modality (STS-IP, STS-S, STS-A) and order of test execution (STS-S and STS-A performed as second or third tests) as fixed effects, and participants as a random intercept, to assess whether order influenced the number of repetitions while accounting for within-subject variability.

To verify inter-evaluator reliability in the STS-A, the ICC, and the Bland and Altman plot were used to verify the association of assessors’ total repetitions. The independent samples *T*-test and Cohen’s *d* effect size were used to compare assessors’ total repetitions. In addition, the Chi-square test was applied to compare the frequency proportions of errors found in the videos: (1) chair size; (2) arms crossed at the sternum without using them to assist in the test; (3) hip touching the seat of the chair; (4) full hip and knee extension; and (5) total test time.

The outlier labeling rule was used to detect outliers and discrepancies (Kannan, Manoj & Arumugam, 2015). Outlier values were calculated by the difference between the 25th and 75th percentiles multiplied by a factor (2.2). The result is then subtracted from the 25th percentile and added to the 75th percentile. The Jamovi statistical program (version 2.4.8; The Jamovi project, Sydney, Australia) and G*Power Statistical Power Analyses software (version 3.1.9.2; Universität Kiel, Germany) were used. Statistical significance was set at 5% (*P* ≤ 0.05; two-tailed).

## Results

### Sample characteristics

There were no dropouts in this study. The sample presented a mean age of 47.8 years and a median time since COVID-19 diagnosis of 11.8 months. The female group presented a significantly lower body mass (75.8 kg *vs.* 93.7 kg) and height (159.2 cm *vs.* 173.0 cm), and a higher FSS score (53.0 *vs.* 21) than the male group (Table 1).

### Synchronous and asynchronous STS tele-assessment validity

STS-IP presented significantly lower total repetitions compared to the STS-S and STS-A (Δ% = −9.6; ES = 0.81, very large, 95% CI [0.44–1.17], *p* < 0.001, and Δ%=−9.8; ES = 0.45, moderate, 95% CI [0.12–0.79]; *p* < 0.001, respectively) (Tables 2 and 3). In addition, STS-IP exhibited significant correlations with STS-S and STS-A repetitions (classified as excellent and good) (ICC = 0.86, 95% CI [0.52–0.95], *p* < 0.001, and ICC = 0.69, 95% CI [0.45–0.83], *p* < 0.001, respectively) (Table 3). The RPE did not differ between any of the STS tests (*p* > 0.05) (Table 2). The STS-S showed an SEM of 2.4 (95% CI [17.4–26.8]) and an MDC of 6.6. Similarly, the STS-A exhibited an SEM of 3.8 (95% CI [14.7–29.6]) and an MDC of 10.5.

**Table 2:** Comparison of repetitions and rating of perceived exertion (RPE) between: (1) STS-IP with STS-S and STS-A and; (2) STS-A with STS-SR. Repetitions are presented as mean (standard deviation) and RPE scores are shown as median (25th and 75th percentiles).

	**Repetitions**	**RPE**
**STS-IP**	20.0 (±6.1)	5.0 (3.0–6.0)
**STS-S**	22.1^*^ (±6.4)	5.5 (4.0–7.0)
**STS-A**	22.1^*^ (±6.8)	5.0 (3.0–7.0)
**STS-SR**	22.9^a^ (±7.2)	5.0 (3.0–7.0)

**Notes.**

* Significant difference from STS-IP (*p* ≤ 0.05).

a Significant difference from STS-A.

STS 1-minute sit-to-stand test A asynchronous RPE rating of perceived exertion S synchronous SR self-reported

**Table 3:** Comparison of intraclass correlation coefficient (ICC), percentage mean difference (Δ%), effect size, confidence interval (95% CI), and Bland and Altman method (mean difference (MD) and range of the interval around the differences (Δ limits)) between STS-IP with STS-S and STS-A repetitions and STS-A with STS-SR.

	**ICC (95% CI)**	**Δ% (Effect size; 95% CI)**	**MD**	**Δ Limits**
**STS-IP *vs.***				
**STS-S**	0.86 (0.52 to 0.95)	−9.6% (0.81; 0.44 to 1.17)	−2.1	10.3
**STS-A**	0.69 (0.45 to 0.83)	−9.8% (0.45; 0.12 to 0.79)	−2.2	18.8
**STS-A *vs.***				
**STS-SR**	0.98 (0.94 to 0.99)	−3.3% (0.59; 0.24 to 0.93)	−0.8	5.0

**Notes.**

STS 1-minute sit-to-stand test A asynchronous S synchronous SR self-reported

**Figure 2:**
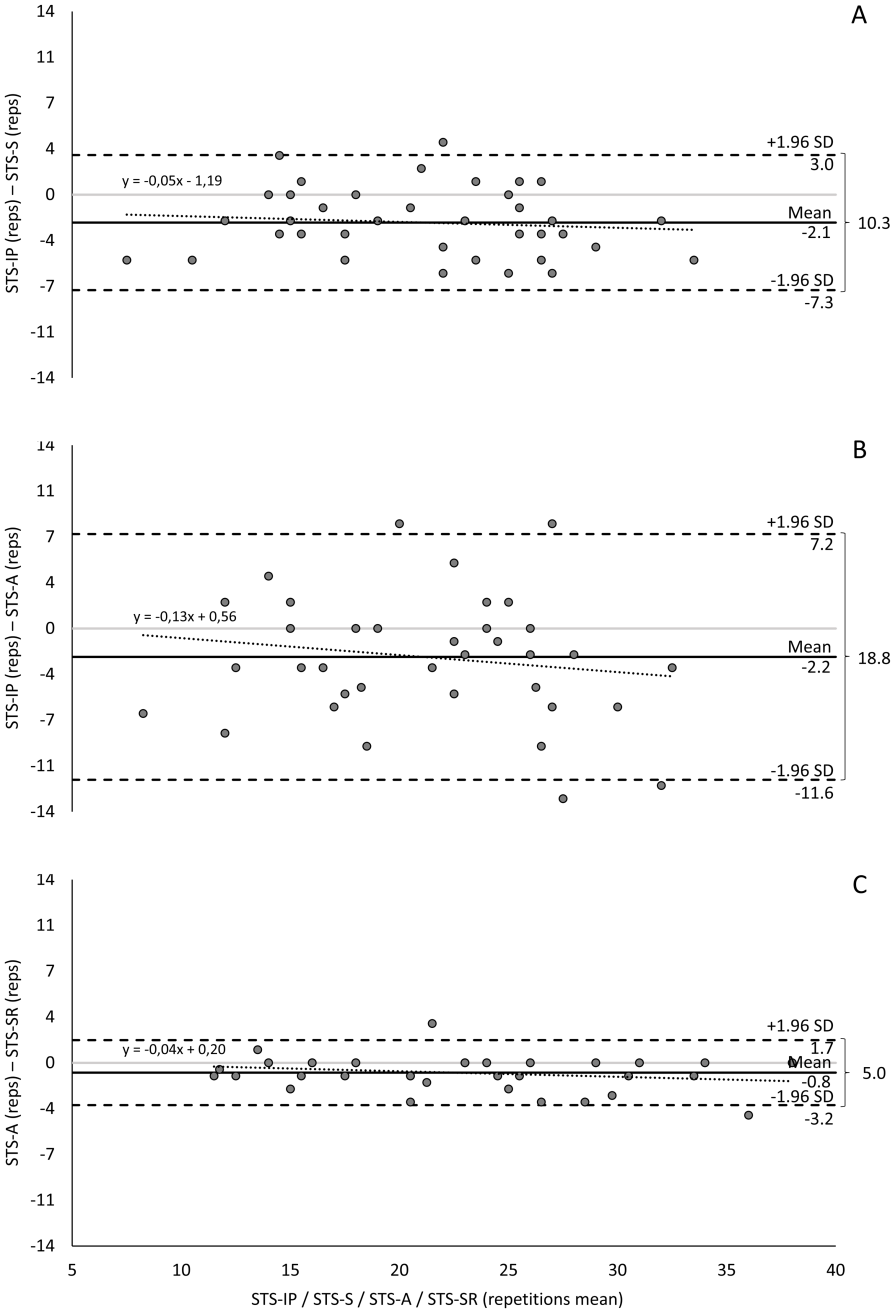
Bland-Altman method comparing STS tele-assessment validity: (A) STS-IP and STS-S; (B) STS-IP and STS-A; and (C) STS-A and STS-SR. The confidence interval was set at 95% (95% CI)±1.96 s.d.: range of the interval around the differences. STS, 1-minute sit-to-stand test; A, asynchronous; S, synchronous; SR, self-reported.

Using the Bland-Altman method, the mean differences between STS-IP with STS-S and STS-A were −2.1 and −2.2 repetitions, respectively. The limits of agreement ranged from −7.3 to 3.0 repetitions and −11.6 to 7.2 repetitions, with two and four data points falling outside these limits, respectively. On average, STS-S underestimated STS-A repetitions by 5% and 13% per additional repetition (Table 3, Fig. 2).

The linear mixed-effects model revealed no significant differences between modalities: STS-S *versus* STS-IP (estimate = 0.12, SE = 1.28, *p* = 0.93) and STS-A *versus* STS-IP (estimate = 0.25, SE = 1.25, *p* = 0.84). However, order had a significant effect on repetitions, with later testing associated with an increase of approximately 1.8 repetitions (estimate = 1.78, SE = 0.70, 95% CI [0.40–3.17], *p* < 0.05).

### Comparisons between STS-A and STS-SR tele-assessment

STS-A repetitions were significantly lower compared to STS-SR (Δ% = −3.3; ES = 0.59, large, 95% CI [0.24–0.93]; *p* < 0.001) (Tables 2 and 3). STS-A repetitions demonstrated significant correlations with STS-SR (classified as excellent) repetitions (ICC = 0.98, 95% CI [0.94–0.99], *p* < 0.001) (Table 3).

The Bland-Altman analysis showed a mean difference of −0.8 repetitions between STS-A and STS-SR, with limits of agreement (±1.96 SD) ranging over 5.0 repetitions (from −3.2 to 1.7 repetitions). Two data points fell outside these limits. On average, STS-A underestimated STS-SR repetitions by 4% for each increment in repetitions (Table 3, Fig. 2).

### Inter-evaluator reliability of the STS-A

There were no significant differences in total repetitions and errors found between evaluators’ assessments of STS-A (Δ% = 1.2; ES < 0.01, trivial, 95% CI [−0.45 to 0.45]; *p* > 0.05) (Table 4). The total repetitions assessed by the evaluators exhibited a significant ICC, classified as excellent (ICC = 0.99, 95% CI [0.99–1.00], *p* < 0.001).

**Figure 3:**
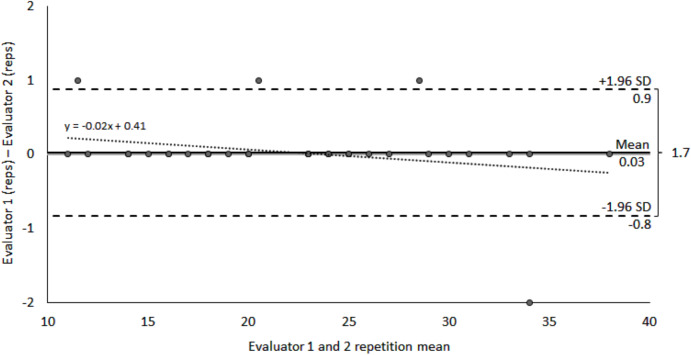
Bland-Altman method comparing STS-A inter-evaluator reproducibility. The confidence interval was set at 95% (95% CI).

The Bland-Altman analysis revealed a difference of 0.03 repetitions between evaluators, with limits of agreement (±1.96 SD) of 1.7 repetitions, extending from −0.8 to 0.9 repetitions. Four data points fell outside these limits. A 2% difference between evaluators was observed for each test repetition (Table 4, Fig. 3).

## Discussion

The objectives of the current study were to validate synchronous and asynchronous STS tele-assessments in individuals with post-COVID-19 condition who did not require ventilator support, analyze the inter-evaluator reliability of the asynchronous STS, and identify the relationship between participants’ self-reported asynchronous STS repetitions and the evaluators’ results. The STS-IP assessment demonstrated significantly lower total repetitions compared to the STS-S and STS-A (approximately 10%), but significant correlations were found between the different tests, indicating the impact of the assessment protocol on STS performance interpretation. Therefore, three comparisons demonstrated excellent agreement, and one (STS-IP *vs.* STS-A) showed good reliability, narrowly below the COSMIN threshold (ICC > 0.70) (Mokkink et al., 2012). Furthermore, the analysis of the relationship between participants’ self-reported asynchronous STS repetitions and the evaluators’ results revealed significant correlations (3.3% lower), suggesting that self-reported values may be feasible and useful in remote settings. Finally, the inter-evaluator reliability was high for the asynchronous STS assessment, with no significant differences in total repetitions or errors found, supporting its practicality and usability in remote settings. These findings provide valuable insights for the validation and implementation of STS tele-assessments in individuals with post-COVID-19 condition.

**Table 4:** Comparison of assessments by Evaluators 1 and 2 for STS-A. The following measures were used to compare the evaluators’ repetitions: ICC (Intraclass correlation coefficient), percentual mean difference (Δ%), effect size, confidence interval (95%CI), and Bland and Altman method (mean difference and range of the interval around the differences (Δ limits)). Repetitions and Total Test Time are presented as mean (standard deviation), and errors found are shown as absolute values (frequency).

	**Evaluator 1**	**Evaluator 2**
**Repetitions**	22.2 (±6.8)	22.1 (±6.9)
***ICC (95% CI)***	0.99 (0.99 to 1.00)	
**Δ*% (Effect size; 95% CI)***	1.2% (<0.01; −0.45 to 0.45)	
***Mean Difference***	0.03	
**Δ* Limits***	1.70	
**Errors found**		
***Chair size***	0 (0.0%)	1 (2.6%)
***Arms crossed***	4 (10.5%)	4 (10.5%)
***Hip touching the seat***	1 (2.6%)	0 (0.0%)
***Hip and knee extension***	2 (5.3%)	8 (21.1%)
***Total test time (sec)***	59.3 (±3.5)	59.3 (±3.5)

**Notes.**

No significant differences were found between evaluators.

Tele-assessment is of paramount importance as it significantly enhances access to assessment services, particularly in situations where distance and transportation hinder in-person assessment. Thus, tele-assessment ensures continuity of care, enables remote monitoring and early intervention, and facilitates efficient research and data collection during a rehabilitation process (Gomes Costa, Ribeiro Neto & Winckler, 2023). While telemedicine has emerged as a powerful tool for post-hospital discharge surveillance in managing patients after COVID-19 (Bartczak et al., 2022), there is a lack of studies validating tele-assessment for evaluating the strength of individuals with post-COVID-19 conditions. Assessing muscle strength, functional capacity, and cardiorespiratory performance in these patients is essential to estimate functional implications, impairments, and exertional desaturation (Belli et al., 2020; Simonelli et al., 2020). In this context, STS-S tele-assessment presented good validity, and it is an alternative tool to facilitate continuous assessments in individuals with post-COVID-19 condition who did not require ventilator support. The STS-S tele-assessment exhibited a 10% higher repetition count compared to the in-person assessment, suggesting a potential learning effect. This finding, combined with an SEM of 2.4 repetitions, implies that considering the MDC of approximately seven repetitions would provide a more accurate approach for interpreting interventions. Therefore, implementing a synchronous familiarization process could lead to more reliable outcomes in STS assessments. Future studies should focus on investigating the specific impact of familiarization on STS-S results.

When analyzing asynchronous tele-assessment, it is important to consider the advantages, such as the ability to save exercise data even after an internet disconnection and resume the assessment once the connection is restored. Additionally, asynchronous tele-assessment may be more cost-effective for participants and requires less time from the telecoach (Lai et al., 2016). However, it is worth noting that asynchronous tele-assessment tends to yield lower validity of results compared to synchronous tele-assessments (Gomes Costa et al., 2022; Gomes Costa, Ribeiro Neto & Winckler, 2023), a trend also observed in our study. Although STS-A average repetitions were similar to STS-S, the correlation with STS-IP was lower and the SEM higher (3.8 reps), suggesting a larger MDC (≈11 repetitions) is needed to detect meaningful changes. These differences may be due to higher variability, with errors such as arms not crossed at the sternum and incomplete hip/knee extension noted in video analysis. It is also important to emphasize that, according to COSMIN standards (Mokkink et al., 2012), SEM and MDC should ideally be derived from test–retest data within the same method under stable conditions, reflecting true measurement error. Our design did not allow for this estimation; therefore, SEM and MDC values were calculated for STS-S and STS-A and should be interpreted as agreement indices between modalities rather than indicators of reliability for longitudinal tracking. Additionally, the linear mixed-effects model indicated that learning or fatigue effects related to order may bias performance outcomes by approximately 1.8 repetitions, whereas modality itself did not significantly affect the number of repetitions. Despite these issues, favorable Bland-Altman results, excellent correlation, and small relative evaluator differences support STS-A reliability and moderate evidence of validity as a tele-assessment tool. Moreover, STS-A reduces test duration, participant costs and enables the evaluation of more participants in less time. These findings allow professionals to choose appropriate strength assessments while weighing limitations and benefits.

The final STS tele-assessment analyzed was the self-reported assessment, which has the advantage of not requiring video analysis, allowing for tele-assessments of larger samples in less time. However, there is a higher risk of errors, which consequently reduces the validity of the results. Similar to a previous study (Gomes Costa et al., 2022), the participants’ self-reported asynchronous STS showed a significant correlation with the STS-A repetitions, presenting an overestimation of 3.3% of the total repetitions (5). The same explanation for the lower repetition results of the STS-A may be applied to understand participants’ underestimation of total repetitions in the STS-SR. The participants registered all repetitions, even those that were not valid, and the result was compared to the correct asynchronous tele-assessment as assessed by the two evaluators. The authors emphasize the necessity to better explain the criteria of STS in order to perform the STS-A and STS-SR with reduced measurement error. The STS-SR presents limitations (overestimated results), and adjusting the test explanations may lead to a more accurate tele-assessment in the context of overcoming distance and time limitations.

The present study showed that the STS results in post-COVID-19 participants ranged from 20 to 23 repetitions. These findings align with previous studies involving patients with similar characteristics (45 to 55 years), where females and males demonstrated 19 and 21 repetitions, respectively, and individuals recovering from COVID-19 pneumonia, with a mean age of 63 years, achieved 21 repetitions (Núñez-Cortés et al., 2021). Furthermore, our results surpassed the STS performance of patients using oxygen (mean age of 74 years) with nine repetitions (Zampogna et al., 2021) and individuals with post-COVID-19 condition symptoms (mean age of 62 years) with 16 repetitions (Baricich et al., 2021). However, it is important to note that our STS results were approximately 50% of the reference values established for a non-clinical population within the same age range (45 to 55 years), where females and males achieved 41 and 44 repetitions, respectively (Strassmann et al., 2013). Additionally, when considering Brazilian reference values for younger adults (aged 40 to 49 years), women and men performed, on average, 31 and 34 repetitions (Furlanetto et al., 2022), respectively, which still represent substantially higher values than those observed in our sample. These variations in performance highlight the heterogeneity of results influenced by factors such as age, sex, and COVID-19 symptoms, underscoring the need for future reference values specifically tailored to this population.

Overall, the present study contributes to the validation and understanding of synchronous and asynchronous STS tele-assessments in individuals with post-COVID-19 condition. The results emphasize the importance of considering the assessment protocol and evaluating inter-evaluator reliability when implementing STS tele-assessments. Additionally, the findings support the value of self-reported repetitions as a useful adjunct to objective evaluators’ assessments. These insights have practical implications for the remote monitoring and management of individuals with post-COVID-19 condition, providing valuable information for clinicians and researchers in this context.

### Study limitations

Firstly, no scale of recuperation was used prior to the tests to confirm if the participants were in the same condition for testing. Standard interval times of 48 to 72 h were employed with the expectation that this potential confounder would not significantly affect the test results. Secondly, the use of non-invasive ventilation or oxygenation strategies was not controlled for, which could introduce a potential source of variability in the STS repetitions. Additionally, the in-person assessment was always conducted first, primarily to ensure patient safety by allowing verification of proper test execution and monitoring of physiological parameters. However, this fixed order may have introduced a learning effect, as the analysis indicated that order biased performance outcomes by approximately 1.8 repetitions. Moreover, although the sample size was calculated to ensure sufficient power for the main analyses and is classified as “fair” according to COSMIN standards (Mokkink et al., 2012), a larger sample would have improved statistical power, allowed for subgroup analyses (*e.g.*, by sex), and elevated the methodological quality classification to “good” or “excellent.” Lastly, the sample included both women and men, and due to the sample size, sex-based strength differences were not controlled for. Further studies that consider fatigue and strength assessments, and that stratify the analysis by sex, may provide new insights into STS in individuals with post-COVID-19 condition who did not require ventilator support. Finally, the calculation of SEM and MDC in this study does not align with COSMIN standards for test–retest reliability, since they were derived from comparisons between different methods rather than repeated measures under stable conditions. Consequently, these values should not be used to interpret longitudinal changes in individual patients. Instead, they should be viewed as agreement metrics between modalities, providing insight into the precision of equivalence between in-person and remote assessments. Future studies employing true test–retest designs are needed to establish measurement error and minimal detectable change within each assessment mode.

## Conclusion

The present study demonstrated good and moderate evidence of validity for synchronous and asynchronous remote STS assessments, in individuals with post-COVID-19 condition who did not require ventilator support, highlighting the impact of the assessment protocol on STS performance interpretation. The findings showed significant correlations between different tests, indicating the potential utility of self-reported repetitions as a remote assessment. The inter-evaluator reliability of asynchronous STS was high, supporting its practicality and usability in remote settings. However, it is important to acknowledge the limitations of asynchronous tele-assessment, such as lower validity results compared to synchronous assessments. The findings provide valuable insights for the validation and implementation of STS tele-assessments in individuals with post-COVID-19 condition who did not require ventilator support, contributing to the understanding and management of this population in remote settings.

##  Supplemental Information

10.7717/peerj.20211/supp-1 Supplemental Information 1 Data

## Additional Information and Declarations

### Data Availability

The following information was supplied regarding data availability: The data is available in the Supplemental File.
